# Molecular basis for the interaction of cellular retinol binding protein 2 (CRBP2) with nonretinoid ligands

**DOI:** 10.1016/j.jlr.2021.100054

**Published:** 2021-02-23

**Authors:** Josie A. Silvaroli, Jacqueline Plau, Charlie H. Adams, Surajit Banerjee, Made Airanthi K. Widjaja-Adhi, William S. Blaner, Marcin Golczak

**Affiliations:** 1 Department of Pharmacology, School of Medicine, Case Western Reserve University, Cleveland, OH, USA; 2 Department of Chemistry and Chemical Biology, Cornell University, Ithaca, NY, USA; 3 Northeastern Collaborative Access Team, Argonne National Laboratory, Argonne, IL, USA; 4 Department of Medicine, College of Physicians and Surgeons, Columbia University, New York, NY, USA; 5 Cleveland Center for Membrane and Structural Biology, School of Medicine, Case Western Reserve University, Cleveland, OH, USA

**Keywords:** lipid transport, retinoids, lipid transfer proteins, retinol binding protein, monoacylglycerols, cellular retinol binding protein 2, *Rbp2*, acyl chain, high-throughput screening of lipids, AEA, arachidonyl ethanolamine, 1-AG, 1-arachidonoylglycerol, 2-AG, 2-arachidonoylglycerol, 3-AG, 3-arachidonoylglycerol, 2-AGE, 2-arachidonoyl glycerol ether, 1-ASG, arachidonoyl-1-thioglycerol, 3-ASG, arachidonoyl-3-thioglycerol, atROL, all-trans-retinol, CRBP2, cellular retinol binding protein 2, FABP1, fatty acid binding protein 1, GIP, gastric inhibitory polypeptide, HTS, high-throughput screening, 2-LaG, 2-lauroyl-glycerol, 2-LG, 2-linoleoyl glycerol, MAGs, monoacylglycerols, 2-OG, 2-oleoyl glycerol, PDB, Protein Data Bank, 2-PG, 2-palmitoyl glycerol

## Abstract

Present in the small intestine, cellular retinol binding protein 2 (CRBP2) plays an important role in the uptake, transport, and metabolism of dietary retinoids. However, the recent discovery of the interactions of CRBP2 with 2-arachidonoylglycerol and other monoacylglycerols (MAGs) suggests the broader involvement of this protein in lipid metabolism and signaling. To better understand the physiological role of CRBP2, we determined its protein-lipid interactome using a fluorescence-based retinol replacement assay adapted for a high-throughput screening format. By examining chemical libraries of bioactive lipids, we provided evidence for the selective interaction of CRBP2 with a subset of nonretinoid ligands with the highest affinity for sn-1 and sn-2 MAGs that contain polyunsaturated C18-C20 acyl chains. We also elucidated the structure-affinity relationship for nonretinoid ligands of this protein. We further dissect the molecular basis for this ligand's specificity by analyzing high-resolution crystal structures of CRBP2 in complex with selected derivatives of MAGs. Finally, we identify T51 and V62 as key amino acids that enable the broadening of ligand selectivity to MAGs in CRBP2 as compared with retinoid-specific CRBP1. Thus, our study provides the molecular framework for understanding the lipid selectivity and diverse functions of CRBPs in controlling lipid homeostasis.

The physicochemical properties of small lipophilic molecules, such as retinoids, long-chain fatty acids, or monoacylglycerols (MAGs), prevent them from partitioning into the aqueous milieu of the cytosol. Therefore, the cellular uptake, intracellular trafficking, signaling, and metabolism of these lipophilic compounds are facilitated by specialized carrier proteins. One of the largest classes of lipid binding proteins are lipocalins (1, 2, 3). These widespread and diverse proteins provide a uniform scaffold that can be adapted to provide specificity for a variety of lipid substrates. Because of their high affinity for all-*trans*-retinol (atROL) and all-*trans*-retinal, a subclass of mammalian intracellular lipocalins has been designated as cellular retinol binding proteins (CRBPs) (4, 5).

Four CRBPs can be found in humans, encoded by the *RBP1*, *RBP2*, *RBP5*, and *RBP7* genes (4, 5). They differ in tissue distribution, expression levels, and affinity for ROL. Thus, they exhibit nonredundant biological functions in controlling the intracellular transport and metabolism of retinoids (5, 6). For example, extensively studied CRBP2 (encoded by the *RBP2* gene) is expressed exclusively in the small intestine (7). The phenotypic characterization of *Rbp2*^−/−^ mice established that this protein plays an essential role in facilitating the uptake and metabolism of ROL by enterocytes, particularly when the dietary availability of vitamin A is limited (8). Interestingly, the recent discovery of the interactions of CRBP2 with 2-arachidonoylglycerol (2-AG) and related MAGs added another dimension to the well-established retinoid-dependent physiological function of CRBP2 (9). It has been shown that in the absence of CRBP2, mice were more susceptible to developing obesity and metabolic disorders. This phenotype was associated with variety of metabolic abnormalities, including decreased energy expenditure, elevated levels of small intestinal MAGs, impaired response to glucose challenge, and an altered regulation of gastric inhibitory polypeptide (GIP) synthesis and/or release from enteroendocrine cells. *Rbp2*^−/−^ mice maintained normal serum retinol levels. Also, there were no genotype-dependent differences in all-*trans*-retinoic acid levels in the liver, white adipose tissue, or fasting and nonfasting intestinal tissue (9). These findings clearly indicate the previously unanticipated role of CRBP2 in energy homoeostasis on both the small intestinal and systemic levels. However, the molecular mechanism by which CRBP2 acts in intestinal enteroendocrine signaling remains unclear.

The key to elucidating the physiological function of CRBP2 is a better understanding of the lipid binding selectivity of this protein. All CRBPs share the same protein structure architecture. These small soluble proteins fold into a single β-barrel that shields the central hydrophobic cavity that constitutes a solitary binding site for retinoids (9, 10, 11, 12). Access to the binding pocket is governed by a “portal region” composed of conformationally flexible loops connecting neighboring β-strands (Fig. 1A) (13, 14). The specific contact sites of a ligand inside the binding cavity, as well as strong interactions with the side chains of the portal region, were shown to be decisive for binding affinity (15).

**Fig. 1:**
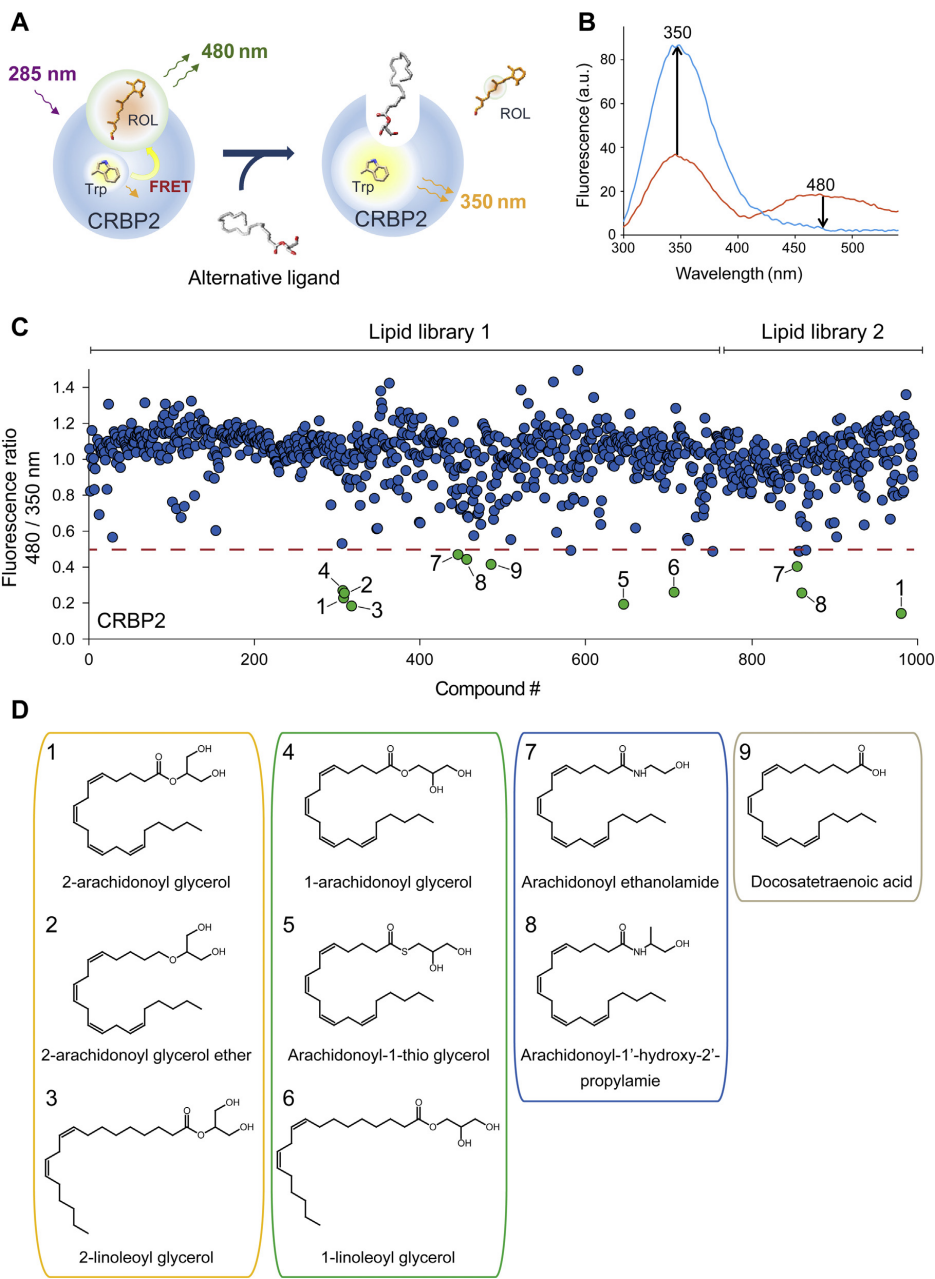
The basis and results of the high-throughput screening for nonretinoid ligands of cellular retinol binding protein 2 (CRBP2). A: A schematic representation of the vitamin A-displacement assay. Reduction of fluorescence resonance energy transfer between the protein scaffold and all-*trans*-retinol (atROL) moiety is used as a measure of the replacement or liberation of atROL from holo-CRBP2. B: As atROL exits the binding site, the fluorescence signal at 350 nm increases, whereas retinoid fluorescence at 480 nm declines. The ratio between the signal at 480 and 350 nm provided a reliable assay readout. C: The result of the primary screening of a chemical library composed of bioactive lipids. D: Chemical structures of nine high-throughput screening hits that corresponded to four different classes of lipids.

CRBP2 is the first and only member of the CRBP class that has been shown to bind endogenous lipids other than ROL or retinal. Moreover, previous studies indicated that CRBP1, a close homologue of CRBP2, has a very narrow ligand specificity essentially limited to retinoids and drug-like xenobiotics (11, 12). Thus, the dogma-shifting observation of the binding of CRBP2 with MAGs raises fundamental questions regarding the types of molecules that can interact with CRBP2, the chemical boundaries that define the specificity of these interactions, and the adaptations of the protein scaffold that enable the alternative ligand specificity. Answering these questions is a necessary step toward unveiling the complex role of CRBP2 and other CRBPs in lipid uptake, transport, and metabolism.

Therefore, to map the lipid interactome of CRBP2, we undertook the unbiased approach of screening a chemical library of diverse endogenous lipids. We established a structure-affinity relationship for the identified compounds and determined the molecular basis for the protein-ligand interactions by solving high-resolution crystal structures for human CRBP2 in complex with selected ligands. Finally, we identified key residues responsible for the differences in the lipid binding selectivity observed in the CRBP protein family. Our data provide a molecular framework for a better understanding of the diverse functions of CRBPs in controlling lipid homeostasis.

## Materials and methods

### Expression and purification of CRBP2

Purification of human CRBP2 was performed essentially as described previously (9). The synthetic clone of cDNA (NP_004155.2; Origene Technologies) was inserted into pET3b vector (Novagen). Plasmid was then transformed into *Escherichia coli* BL21(DE3) strain (New England Biolabs). Bacteria were grown in lysogeny broth media in the presence of 50 μM kanamycin at 37°C in a shaker incubator. The protein expression was induced with 0.5 mM isopropyl β-d-1 thiogalactopyranoside (Roche) at an absorbance of 0.6–0.8. Bacteria were grown for an additional 4 h and harvested by centrifugation (6,000 *g*, 15 min, 4°C). Cells were lysed by osmotic shock, sonification, and centrifuged (36,000 *g*, 30 min, 4°C). Supernatant was collected, adjusted to 50 mM Tris-HCl (pH 8.0), 250 mM NaCl, and loaded onto a nickel-nitrilotriacetic acid agarose resin column (GE Healthcare) that had been washed with 100 ml of 50 mM Tris-HCl (pH 8.0), 250 mM NaCl, and 5 mM imidazole. Bound CRBP2 was eluted by raising imidazole concentration to 250 mM. Eluted fractions were pooled together and concentrated to 5 ml. The solution was then diluted 40-fold with 10 mM Tris-HCl (pH 8.0) and loaded onto a 5 ml HiTrap Q HP ion exchanger column (GE Healthcare). CRBP2 was eluted in a linear gradient of NaCl 0–1 M in 10 mM Tris-HCl buffer (pH 8.0). The molecular identification and purity of the purified protein was examined by LC/MS. Fractions containing purified protein were collected and concentrated to 3 mg/ml and stored at −80°C. Folch's extracts of purified recombinant CRBP2 (1 mg) did not reveal the presence of fatty acids or MAGs detectable by LC/MS (9, 16). Also, the incubation of purified apo-CRBP2 with atROL led to efficient formation of atROL-CRBP2 complex with 1:1 ligand to protein stoichiometry as verified by UV/visible absorbance and HPLC quantification of the retinoid content (7, 9). Therefore, the purification protocol did not include a further delipidation step.

### Reconstitution of CRBP2-atROL complex

To prepare holo-CRBP2, 2 mg of purified apoprotein was incubated for 20 min on ice in 10 mM Tris-HCl, pH 8.0%, 10% glycerol (v/v) with ∼2 M excess of atROL (Toronto Research Company) delivered in ethanol (1%; v/v). To remove unbound retinoids, the protein solution was diluted tenfold with 10 mM Tris-HCl, pH 8.0, centrifuged to remove precipitates (15,000 *g*, 5 min, 4°C), and loaded onto a 5 ml HiTrap Q HP column. Holo-CRBP2 was eluted from the column as described above. The efficiency of holo-CRBP2 formation was examined spectrophotometrically by recording UV/visible spectrum. The complex of CRBP2 with atROL revealed characteristic two absorbance maxima at 280 and 348 nm with ratio ∼1.3 (7).

### Mutagenesis of *Crbp2*

To introduce substitute mutations T51/I and V62/M, cDNA of CRBP2 was cloned into pCR 2.1 vector (Thermo Fisher Scientific). The mutations were introduced by PCR using 5’ phosphorylated primers of the following sequences: T51/I: forward, AACTTCAAGATTAAAACCACTAGCACATTCC; reverse, ATCACCATCTTGATCAATAACCTTCGTCTG; V62/M: forward, CGCAACTATGATATGGATTTCACTGTTGG; reverse, GAATGTGCTAGTGGTTTTTGTCTTGAAGTTATCACC. The presence of desired substitutions was verified by sequencing. To obtain mutated proteins, their cDNAs harboring these changes were cloned into pET3b vector and expressed and purified as described above.

### High-throughput screening for CRBP2 ligands

The high-throughput screening (HTS) was similar to previously published method (12). HTS was performed in 96-well format using UV transparent plates (Corning). Two hundred microliters of a buffer containing 20 mM Tris-HCl (pH 7.4), 250 mM KIO_3_, 5% glycerol, and 0.5 μM CRBP2 filled each well. Lipid libraries from Cayman Chemicals (Bioactive Lipid I Screening Library) and Enzo Life Science (Screen-Well Bioactive Lipid Library) were screened by pipetting 1 μl of each compound (dissolved in DMSO) into a well to a final concentration of 5 μM. Using Flexstation 3 microplate, plates were excited at 285 nm, and fluorescence was recorded at 350 and 480 nm. The ratio between the fluorescence intensity at 480 and 350 nm (F_480_/F_350_) were calculated for each examined compound. The threshold for a positive HTS hit was set at the F_480_/F_350_ value of 0.5 to allow for detection of ligands with moderated (low micromolar range) or lower affinity for CRBP2. Compounds that caused increase of the fluorescence signal at 350 nm without concomitant decrease of the fluorescence at 480 nm or vice versa were considered false positive.

### Determination of binding affinity for nonretinoid ligands

The chromophore replacement assay was performed as described by Silvaroli *et al.* (12). CRBP2 in complex with atROL was excited with photons at a wavelength of 285 nm. Fluorescence emission was monitored at 350 and 480 nm. Titration with an alternative ligand causes liberation of atROL from the binding pocket and lack of fluorescence resonance energy transfer between the tryptophan residues and the retinoid moiety. The net effect is decrease in the fluorescence signal at 480 nm and concomitant increase at 350 nm (Fig. 1A, B). The experiments were performed on PerkinElmer Life Science LS55 spectrofluorometer at 25°C, using a 1 cm quart cuvette. Three microliters of buffer, 67 mM phosphate-buffered saline (pH 7.4%), 5% glycerol (v/v) containing 1 μg/ml of CRBP2 were dispensed into the cuvette. Increasing concentrations of tested compounds were delivered in acetonitrile. The final concentration of the organic solvent did not exceed 1% (v/v). The obtained fluorescence spectra corrected for the background. Analysis of the changes in the maximum emission values at the different concentrations of ligands was used to calculate *K*_d_ values using the saturation single ligand binding model (SigmaPlot 11 software package; Systat Software). Each *K*_d_ listed in Table 1 represents an average value obtained from at least three independent titrations.

**Table 1:** Nonretinoid lipids and their affinity for human CRBP2

Structure	Name	Abbreviation	Alkyl Chain Length (Carbon Atoms)	Double Bonds	*K*_d_ (nM)	Crystal Structure (PDB no.)
	2-Arachidonoyl glycerol	2-AG	20	4	27.1 ± 2.5 (9)	6BTH (9)
	1-Arachidonoyl glycerol	1-AG	20	4	37.9 ± 3.3 (9)	7JVG
	2-Linoleoyl glycerol	2-LG	18	2	40.0 ± 4.4 (9)	7JWD
	1-Linoleoyl glycerol	1-LG	18	2	97.1 ± 27.6	-
	2-Oleoyl glycerol	2-OG	18	1	65.4 ± 4.4 (9)	7JWR
	1-Arachidoyl glycerol	1-ArG	20	0	132.6 ± 33.1	-
	2-Stearoyl glycerol	2-SG	18	0	54.8 ± 9.8	-
	2-Palmitoyl glycerol	2-PG	16	0	51.2 ± 7.7	7JX2
	2-Lauroyl glycerol	2-LaG	12	0	172.4 ± 8.8	7K3I
	1-Decanoyl glycerol	1-DG	10	0	779.8 ± 86.5	-
	2-Arachidonoyl glycerol ether	2-AGE	20	4	45.7 ± 2.7	7JVY
	Arachidonoyl-1-thio-glycerol	1-ASG	20	4	89.3 ± 14.3	7JZ5
	1-O-hexadecyl glycerol	1-O-HG	16	0	153.0 ± 7.3	-
	Arachidonoyl ethanolamide	AEA	20	4	663.0 ± 24.0 (9)	6BTI (9)
	Docosatetraenoic acid	ADA	22	4	217.4 ± 14.4	-

### Crystallization of CRBP2 in complex with lipids and X-ray diffraction data processing

The crystallization conditions were identical to those published by Lee *et al.* (9). Human CRBP2 in complexes with nonretinoid ligands were crystallized using the sitting drop vapor diffusion method. Prior to setting up crystallization drops, CRBP2 (9 mg/ml) was incubated with 50 μM of ligand in 10 mM Tris-HCl (pH 8.0) and 150 mM NaCl for 20 min on ice. Protein samples were mixed in a 1:1 ratio with 1 μl of 0.1 M Tris-HCl (pH 8%) and 22–28% polyethylene glycol 3350. The wells were sealed and stored at room temperature. Crystals were collected, cryoprotected in 10% glycerol, 10% polyethylene glycol 2000 (v/v), and flash frozen in liquid nitrogen.

X-ray diffraction data were collected at the Advanced Photon Source Northeastern Collaborative Access Team 24-ID-C and 24-ID-E beamlines. Data reduction was performed using iMosflm software package (17). The structures were solved by molecular replacement with PHASER_MR (18) and the refined model of CRBP2 [Protein Data Bank (PDB) accession code: 6BTI] as a search model. Adjustments to the protein model were made with COOT (19), and the initial model was refined with PHENIX (20). X-ray data collection and refinement statistics can be found in Table 2. The coordinates and structure factors for each of the structure analyzed in this report were deposited in the PDB under the following accession codes: 7JVG, 7JVY, 7JWD, 7JWR, 7K3I, 7JX2, and 7JZ5 (Table 2).

**Table 2:** X-ray data collection and refinement statistics

Protein	CRBP2						
Ligand	1-AG	2-AGE	2-LG	2-OG	2-LaG	2-PG	1-ASG
PDB accession code	7JVG	7JVY	7JWD	7JWR	7K3I	7JX2	7JZ5
Beamline	24-ID-C	24-ID-C	24-ID-C	24-ID-C	24-ID-E	24-ID-C	24-ID-C
Wavelength (Å)	0.9791	0.9791	0.9791	0.9791	0.9791	0.9791	0.9791
Data collection							
Space group	*P*2_1_2_1_2_1_	*P*2_1_2_1_2_1_	*P*2_1_2_1_2_1_	*P*2_1_2_1_2_1_	*P*2_1_2_1_2_1_	*P*2_1_2_1_2_1_	*P*2_1_2_1_2_1_
Cell dimensions *a*, *b*, *c* (Å)							
	57.9, 67.4, 88.9	57.9, 67.7, 88.8	57.7, 67.7, 88.8	58.1, 67.4, 88.0	37.3, 64.6, 67.0	37.3, 64.4, 66.6	57.8, 67.6, 89.5
Resolution (Å)	67.43–1.40 (1.42–1.40)^a^	53.82–1.30 (1.32–1.30)^a^	57.72–1.35 (1.37–1.35)^a^	67.38–1.30 (1.32–1.30)^a^	64.56–1.20 (1.22–1.20)^a^	66.58–1.80 (1.84–1.80)^a^	89.48–1.57 (1.59–1.57)^a^
Rmerge (%)	7.9 (69.6)^a^	7.8 (59.4)^a^	6.1 (29.9)^a^	7.0 (78.6)^a^	7.1 (89.9)^a^	11.3 (87.3)^a^	10.2 (75.8)^a^
Rpim (%)	5.1 (45.6)^a^	5.1 (43.6)^a^	4.0 (21.1)^a^	4.6 (59.7)^a^	4.5 (66.4)^a^	7.2 (55.4)^a^	4.4 (32.2)^a^
*I*/σ*I*	10.6 (2.1)^a^	10.6 (2.1)^a^	14.1 (3.7)^a^	10.9 (1.6)^a^	8.4 (1.5)^a^	8.2 (2.3)^a^	14.1 (2.1)^a^
*CC*(*1*/*2*)	0.99 (0.84)^a^	0.99 (0.84)^a^	0.99 (0.92)^a^	0.99 (0.85)^a^	0.99 (0.67)^a^	0.99 (0.88)^a^	0.99 (0.85)^a^
Completeness (%)	96.7 (95.9)^a^	99.3 (98.7)^a^	93.0 (72.0)^a^	99.6 (62.7)^a^	98.8 (85.6)^a^	99.7 (100)^a^	99.6 (93.8)^a^
Redundancy	6.1 (6.1)^a^	5.9 (5.1)^a^	5.7 (4.9)^a^	5.8 (4.2)^a^	6.4 (5.1)^a^	6.3 (6.6 )^a^	6.5 (6.3)^a^
Refinement							
Resolution (Å)	53.73–1.40	53.82–1.30	48.39–1.35	48.48–1.30	46.50–1.20	46.31–1.80	53.92–1.57
No. of reflections	66,657	85,578	71,165	82,949	50,715	15,383	49,642
*R*_work_/*R*_free_ (%)	15.2/19.6	13.6/16.6	11.7/14.8	13.3/17.4	18.4/20.6	20.6/25.5	16.1/18.3
No. of atoms	2,958	3,013	3.057	3.022	1,576	1,351	2,829
Proteins	2,297	2,323	2,306	2,358	1.183	1,119	2,273
Ligand	54 (1AG)^b^	52 (VKV)^b^	50 (VL7)^b^	50 (YOG)^b^	19 (VL2)^b^	23 (VLP)^b^	54 (VPY)^b^
Water	607	638	701	593	397	209	502
Mean *B*-factor (Å^2^)							
Protein	13.2	13.9	15.0	19.0	18.4	29.2	25.2
Ligand	22.7 (1AG)^b^	24.6 (VKV)^b^	23.5 (VL7)^b^	30.0 (YOG)^b^	23.2 (VL2)^b^	35.0 (VLP)^b^	32.9 (VPY)^b^
Water	31.1	34.2	36.3	38.2	33.9	40.1	38.1
Root-mean-square deviations							
Bond lengths (Å)	0.011	0.009	0.009	0.009	0.007	0.008	0.008
Bond angles (°)	1.270	1.233	0.962	0.977	1.035	0.789	1.034
Validation							
Ramachandran plot							
Fav/outliers (%)	98.1/0	98.5/0	98.2/0	98.6/0	97.9/0	97.7/0	98.2/0
Rotamer outliers (%)	0	0	0	0	0	0	0
Clash score	4.0	4.1	2.8	2.3	2.1	2.7	3.1

Reported data set was collected on a single crystal.

a Highest-resolution shell is shown in parentheses.

b Ligand's accession code.

### LC/MS

The experiments were performed using LTQ Velos dual-pressure linear ion trap mass spectrometer equipped with an electrospray ionization source (Thermo Scientific) and interfaced with an Agilent Technologies 1100 Series HPLC system. To record spectra of intact proteins, 1 μg of the protein was loaded onto a C8 cartridge column (Luna 5 μm, 20 × 2.0 mm; Fenomenex) and eluted with a linear 2–100% gradient of acetonitrile/water over 20 min at a flow rate of 0.2 ml/min. Both solvents contained 0.1% formic acid. Intact protein masses were deconvoluted either by use of ProMass for Xcaliber software (Thermo Scientific) or manually for selected peaks.

### Calculations of crystallographic *B*-factors for refined models

The averages of the equivalent isotropic crystallographic *B*-factors (*B*_*eq*_) for each residue were calculated and normalized with the “z-score normalization” methodology (21), in which:

$$B_{eqx-zscore\left(i\right)}=\left[B_{eqx\left(i\right)}-\langle B_{eq\left(i\right)}\rangle \right]/{\text{s}}_{\left(i\right)}$$

*B*_*eqx-zscore*(*i*)_ is the normalized z-score for residue *x* in the structure *i*, *B*_*eqx*(i)_ is the equivalent isotropic *B*-factors for residue *x*, 〈*B*_*eq*(i)_〉is the average residue equivalent isotropic *B*-factor for structure *i*, and s_(*i*)_ corresponds to the standard deviation among atoms in the structure.

## Results

### Specific subclasses of endogenous lipids interact with CRBP2

The recent discovery of the interactions of CRBP2 with MAGs and *N*-acylethanolamides raised a fundamental question about the chemical boundaries that define suitable ligands for this protein (9). To address this question in an unbiased and systematic manner, we employed a high-throughput assay to screen natural lipids for any potential CRBP2 binding partners. We combined two commercially available libraries, Bio-Active Lipid I Screening (Cayman Chemicals) and Screen-Well Bioactive Lipid (Enzo Life Sciences, Inc) composed of nearly 1,100 unique compounds that represented 19 different classes of lipids (supplemental Table S1). After the elimination of chemicals with spectral properties interfering with the fluorescence assay, the HTS performed on human recombinant CRBP2 revealed nine independent hits. They represented compounds that clustered into four major groups: 1-monoacylglycerols, 2-monoacylglycerols, *N*-acylethanolamides, and fatty acids (Fig. 1C, D). Noticeably, despite belonging to different classes, all these CRBP2-interacting lipids reveal similar structural motifs. They contain a single acyl chain at the hydrophobic end linked to a glycerol or an ethanolamine moiety that constituted the polar side of the molecule. The only exemption was docosatetraenoic acid having a nonesterified carboxyl group. Because of specific composition of the screened chemical libraries limited to bioactive lipids, the length of the acyl moieties found in the HTS hits was restricted to 18–20 carbon atoms. Moreover, these acyl chains were polyunsaturated with two to four double bonds present in their structures. Thus, among the variety of examined nonretinoid natural lipids, CRBP2 revealed a relatively narrow ligand binding selectivity that is limited to lipids of well-defined molecular architecture.

### Structure-affinity relationship for nonretinoid ligands of CRBP2

Although the results of the HTS established the classes of lipids suitable for the interaction with CRBP2, they did not provide quantitative information about their binding affinities. Based on the chemical structures of the ligands, one can speculate that three independent parameters may contribute to the binding: *i*) the length of the acyl chain, *ii*) the number of double bounds, and *iii*) the number and quality of hydrogen bonds formed between the protein and a ligand in the binding site. To gain a better understanding of the structure-function relationship of the CRBP2 ligands, we determined the dissociation constant (*K*_d_) for the compounds found in the HTS and their derivatives with altered lengths and saturation levels of the acyl chains, as well as the number of polar atoms available for the formation of hydrogen bonds (Table 1).

**Fig. 2:**
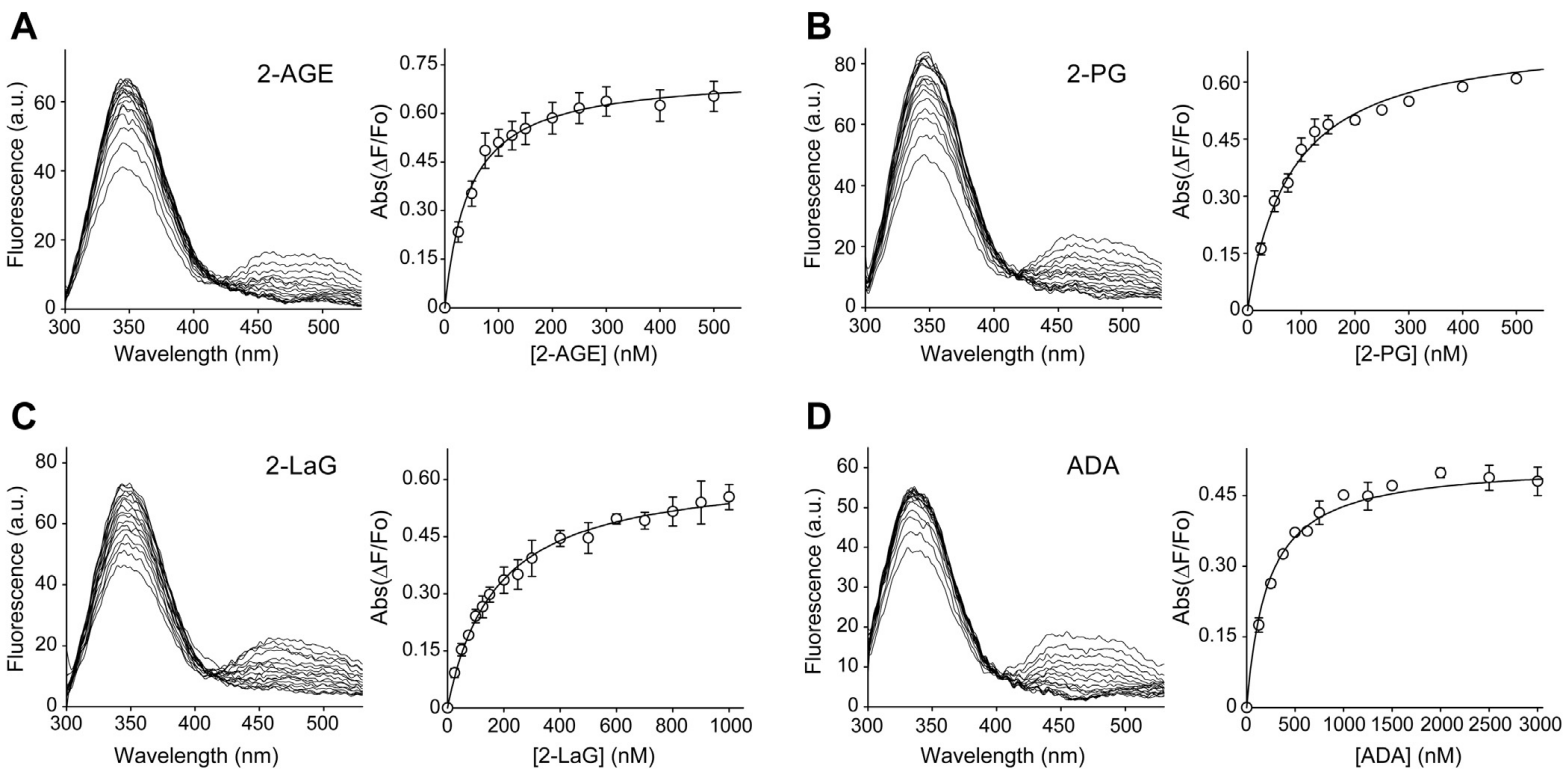
Determination of *K*_d_ values for selected nonretinoid ligands of cellular retinol binding protein 2. Fluorescence spectra of holo-cellular retinol binding protein 2 and corresponding titration curves for (A) 2-arachidonoylglycerol ether (2-AGE), (B) 2-palmitoylglycerol (2-PG), (C) 2-lauroylglycerol (2-LaG), and (D) docosatetraenoic acid (ADA). The experimental data were fitted to the one-site saturation ligand binding model. The *K*_d_ values for these and other examined lipids are summarized in Table 1. The calculations were based on the results from at least three independent experiments for each ligand. Data are presented as mean values ± SD.

Changes in the fluorescence properties of holo-CRBP2 upon titration with the ligands reveal *K*_d_ values that markedly depend on the structural properties of the examined compounds (Fig. 2 and Table 1). The highest affinity was calculated for 2-AG (27.1 ± 2.5 nM) (9). Any deviation for the chemical structure of this compound resulted in a lower binding affinity. As shown previously, a relatively small change was observed for the sn-1 regioisomer of 2-AG. 1-arachidonoylglycerol (1-AG) interacts with CRBP2 with a *K*_d_ of 37.9 ± 3.3 nM. The slight increase in the *K*_d_ value can be attributed to the fact that the 1-AG used in the experiment was a racemic mixture composed of 1-AG ((*S*)-glycerol 1 arachidonate) and 3-arachidonoylglycerol (3-AG) ((*R*)-glycerol 1 arachidonate). In fact, as discussed later in this report, only 1-AG (*S*-isomer) binds to CRBP2. Importantly, an analogous correlation of *K*_*d*_ values was seen for other sn-1 and sn-2 MAG pairs represented by 1-linoleoyl glycerol and 2-linoleoyl glycerol (2-LG) with affinities of 97.1 ± 27.6 and 40.0 ± 4.4 nM, respectively. In this case, a racemic mixture of 1-linoleoyl glycerol/3-LG isomers was also used for the fluorescence titrations.

In contrast, the length of the acyl chain had a strong effect on binding affinity (Table 1). Shortening the chain from 20 to 18 carbon atoms caused a 2-fold decrease in the affinity (*K*_d_ = 54.8 ± 9.8 nM for 2-stearoyl glycerol) as compared with 2-AG. A similar *K*_d_ was measured for 2-palmitoyl glycerol (2-PG; *K*_d_ = 51.2 ± 7.7 nM). However, further shortening the acyl chain to 12 and 10 carbon atoms had a greater effect on binding, lowering the affinity by over 6-fold for 2-lauroyl-glycerol (2-LaG) and 29-fold for 1-decanoyl-glycerol, as compared with 2-AG.

Interestingly, the presence of double bonds within acyl moieties was an important factor for binding affinity. Unsaturated bonds limit the number of configurations that can be adapted by the acyl chains, thus higher number of double bonds correlates with a more defined spatial orientation of the chain. Furthermore, the Z configuration of the double bonds introduces characteristic “kinks” to the carbon backbone. As evident in the crystal structure of CRBP2 in complex with 2-AG (PBD: 6BTH), the rigid configuration of the arachidonoyl moiety played a decisive role in the binding. It adapted an orientation similar to that of the β-ionone ring in atROL (9). Consequently, lowering the number of double bonds present in acyl chains of the same length (C20) resulted in nearly 5-fold decrease in the affinity for racemic 1-arachidonyl glycerol (*K*_d_ = 132.6 ± 33.1 nM). The same trend was observed for C18 chains where the affinity declined from 40.0 ± 4.4 nM for polyunsaturated 2-LG to 65.4 ± 4.4 nM.

Although the size of the MAGs and their prevalence to adapt a bent configuration play an important role in the ligand selectivity of CRBP2, the interaction of the polar atoms of a ligand molecule inside the binding pocket of the protein has the decisive influence on the binding. Even a relatively modest intervention in the atomic composition of hydrogen donors and acceptors involved in the hydrogen bond interactions inflicted a significant decrease in affinities. For example, the elimination of the carbonyl oxygen of the ester group caused a nearly 2-fold increase in *K*_d_ values for 2-arachidonoyl glycerol ether (2-AGE) as compared with 2-AG. The same structure-affinity relationship was observed for MAGs with shorter and saturated acyl chains exemplified by the 2-PG and 1-O-hexadecyl glycerol pair. The lack of the carboxyl oxygen in 1-O-hexadecyl glycerol had a synergistic effect on the affinity, further increasing the *K*_d_ for this compound to 153.0 ± 7.3 nM. The replacement of the oxygen of the ester group with a sulfur atom had a less profound influence on the binding. Arachidonoyl-1-thioglycerol (1-ASG), a thioester derivative of 1-AG, interacted with CRBP2 with an affinity of 89.3 ± 4.6 nM (Table 1). Dramatic change in the affinity was observed for nonesterified docosatetraenoic acid, for which the *K*_d_ was eight times higher than for 2-AG. The affinity was ∼25 times lower when the glycerol backbone was replaced with the ethanolamine moiety as exemplified by arachidonyl ethanolamine (AEA) (*K*_d_ = 663.0 ± 24.0 nM) (9). In conclusion, the above structure-affinity studies clearly demonstrated that CRBP2 reveals binding selectivity toward polyunsaturated MAGs with the highest prevalence for 2-AG endocannabinoid.

### X-ray crystallography reveals structural basis for lipid binding specificity of CRBP2

To better understand the molecular basis for the lipid binding specificity of CRBP2, we obtained crystals and determined high-resolution structures of this protein in complex with seven different ligands used in the structure-affinity studies (Table 2). They represent a selection of 2-AG derivatives with modified molecular properties important for binding to CRBP2 (Table 1). In addition to previously published complexes of CRBP2 with 2-AG and AEA (PDB: 6BTH and 6BTI) (9), these new structures paint an unambiguous picture of the molecule determinants that govern the interaction of CRBP2 with nonretinoid lipids.

### Specificity toward the acyl chains of MAGs

**Fig. 3:**
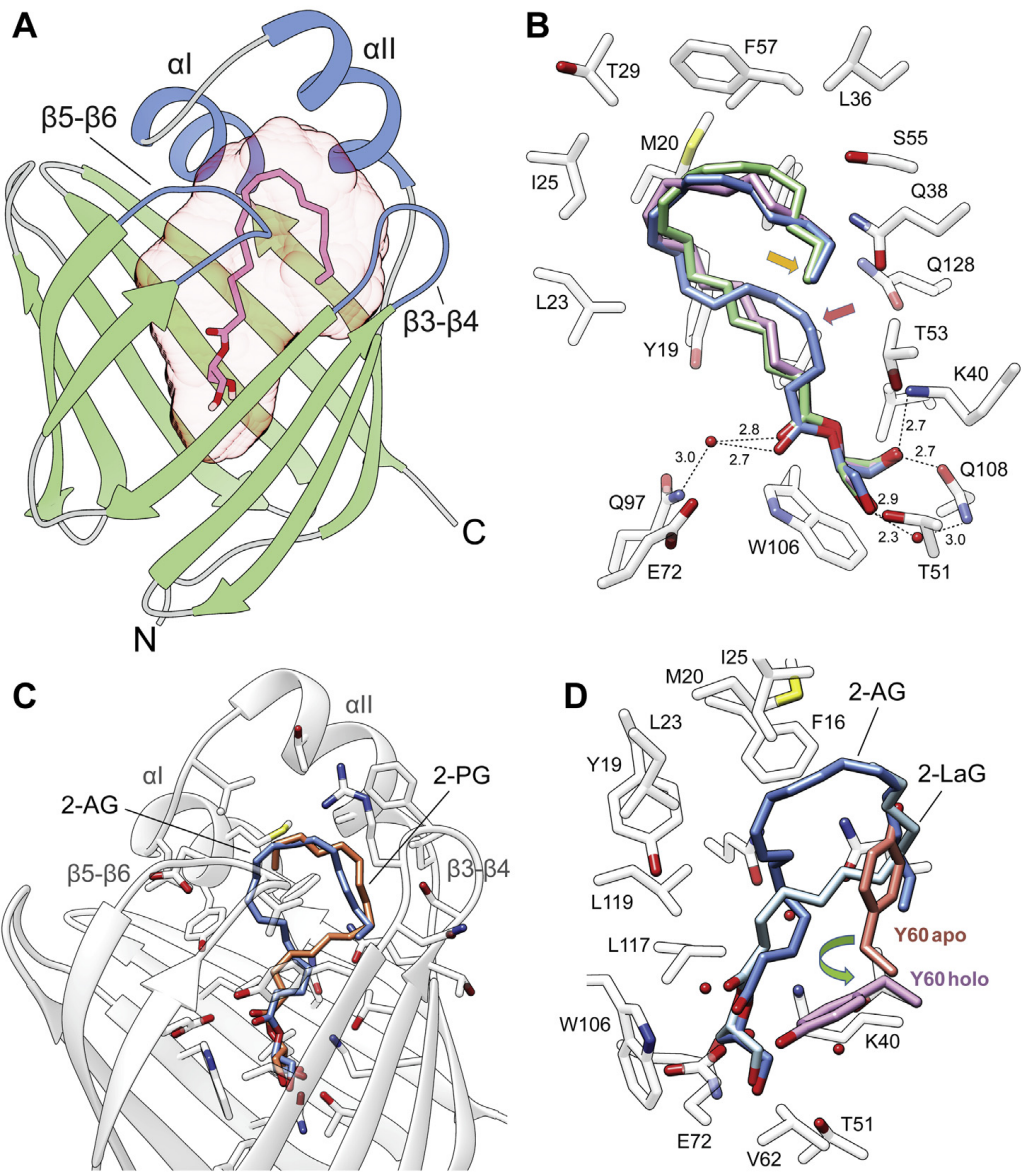
Acyl chain-dependent difference in the binding models of 2-monoacylglycerols to cellular retinol binding protein 2 (CRBP2). A: Ribbon representation of the overlay structures of CRBP2 [Protein Data Bank (PDB): 7JWR]. Position of 2-oleoyl glycerol in the binding pocket (light red transparent surface) is indicated by the model representation of the ligand. The portal region of the protein is colored blue and labeled with corresponding secondary structures. B: Superimposed structures of CRBP2 in complex with 2-arachidonoylglycerol (2-AG) (colored blue) (PDB: 6BTH), 2-oleoyl glycerol (purple) (PDB: 7WR), and 2-linoleoyl glycerol (green) (PDB: 7JWD). The overlapping ends of fatty acid chains are marked with yellow arrow. The difference in the spatial positions of the chain between these monoacylglycerols is marked with red arrow. C: Comparison of the positions of 2-AG (blue) and 2-palmitoylglycerol (2-PG) (orange) (PDB: 7JX2) in the binding site. D: Orientation of the acyl chains of 2-AG and 2-lauroyl-glycerol (2-LaG) (light blue) (PDB: 7K3I) in relation to the side chain of Y60. Regardless the differences in the orientation of the acyl chains inside the binding pocket of CRBP2, both ligands cause repositioning of the Y60 side chain as compared with the apoform of the protein.

An important aspect of the interaction of CRBP2 with MAGs is acyl chain selectivity. Based on the position of 2-AG and the overall size of the binding pocket, it can be inferred that the upper limit for the length of the acyl chain that can be accommodated by the protein is 20 carbon atoms. However, why does the affinity for MAGs with shorter fatty acid moieties decline? As suggested by recent molecular dynamic simulations, the dissociation of atROL from CRBPs depends largely on its interaction with the residues of the portal region, particularly α-helix II and the β3-β4 loop (Fig. 3A) (15). Thus, one can speculate that the interactions between 2-AG and the entry portal site are more favorable than those in MAGs with shorter acyl chains. The comparison of the positions of 2-AG and 2-LG or 2-oleoyl glycerol (2-OG) supports this hypothesis. Despite the two-carbon difference in length, the aliphatic ends of the fatty acid chains for each of these ligands are located exactly in the same place (Fig. 3B). This spatial length compensation is achieved by the adoption of a more extended configuration by the C18 chains of 2-LG and 2-OG as compared with 2-AG. The main difference is seen in the location of carbon atoms 3–6 of the chains, whereas the portions of the fatty acid moieties that make contact with the portal region of the protein are very similar in these ligands. Thus, the conservation of the interactions of lipids with the portal region seems to be key to the stabilization of the protein configuration in the ligand bound (close) state that macroscopically can be observed as lower *K*_d_. However, how can the relatively good binding of 2-PG and 2-LaG be explained if their 16- and 12-carbon acyl chains are too short to interact with the β3-β4 loop? The answer to this question can be determined from the structures of CRBP2 complex in these lipids. They revealed an unexpected change in the orientation of the palmitoyl and lauroyl chains as compared with longer acyl moieties. The acyl chains of 2-AG, 2-LG, or 2-OG bent inside the binding pocket making contact with the side chains of the β5-β6 loop, α-helices I and II, and finally the β3-β4 loop at the ends of the carbon chains. In contrast, the palmitoyl in 2-PG adapted an inverse orientation, twisting first toward the β3-β4 loop before interacting with the α-helices II and I (Fig. 3C). Thus, the interaction with the β3-β4 loop is prioritized, enabling the stabilization of the ligand inside the binding cavity and the protein as whole in the holoconfiguration. The same reverse orientation of the acyl chain is observed in the 4-carbon atoms shorter lauroyl moiety of 2-LaG. Analogously to CRBP1, the incorporation of a lipid into the binding site of CRBP2 is associated with the repositioning of the Y60 side chain that flips outside the binding pocket to make space for the ligand (11, 22). This conformational change is a hallmark of the holo forms of CRBPs. The superimposition of the structures of apo- and 2-PG- or 2-LaG-bound CRBP2 clearly indicates that the inverse orientation of the palmitoyl and lauroyl groups cause the outward rotation of Y60 that would not occur if the position of the acyl chain was similar to that observed for 2-AG (Fig. 3D).

In contrast to the lengths of the fatty acid moieties, comparing the positions of MAGs with polyunsaturated and monounsaturated acyls did not indicate differences in the spatial orientation of the ligands in the binding site. Despite the absence of double bonds, the linoleoyl and oleoyl moieties adopted the same kinked configuration as observed for 2-AG.

The presence of two alternative binding modes that depend on the length of the acyl chains explains the significant decline in the affinity of MAGs with fatty acid moieties shorter than 16 carbon atoms. Although we were not able to obtain diffracting crystals for CRBP2 in complex with 1-decanoyl-glycerol, it is reasonable to speculate that shortening the chains gradually eliminates their ability to interact with the portal region of the protein. The inability to stabilize the close conformation most likely contributes to the rapid diffusion of the ligand out of the binding pocket. This hypothesis is further supported by the comparison of normalized crystallographic *B*-factors calculated for CRBP2 in complex with 2-AG or 2-OG and 2-PG or 2-LaG. The binding of MAGs with shorter and saturated fatty acid chains results in a significant increase in the structural dynamics of α-helix I and β3-β4 loop of the protein's entry port (supplemental Fig. 1).

### Enantioselectivity binding of MAGs

**Fig. 4:**
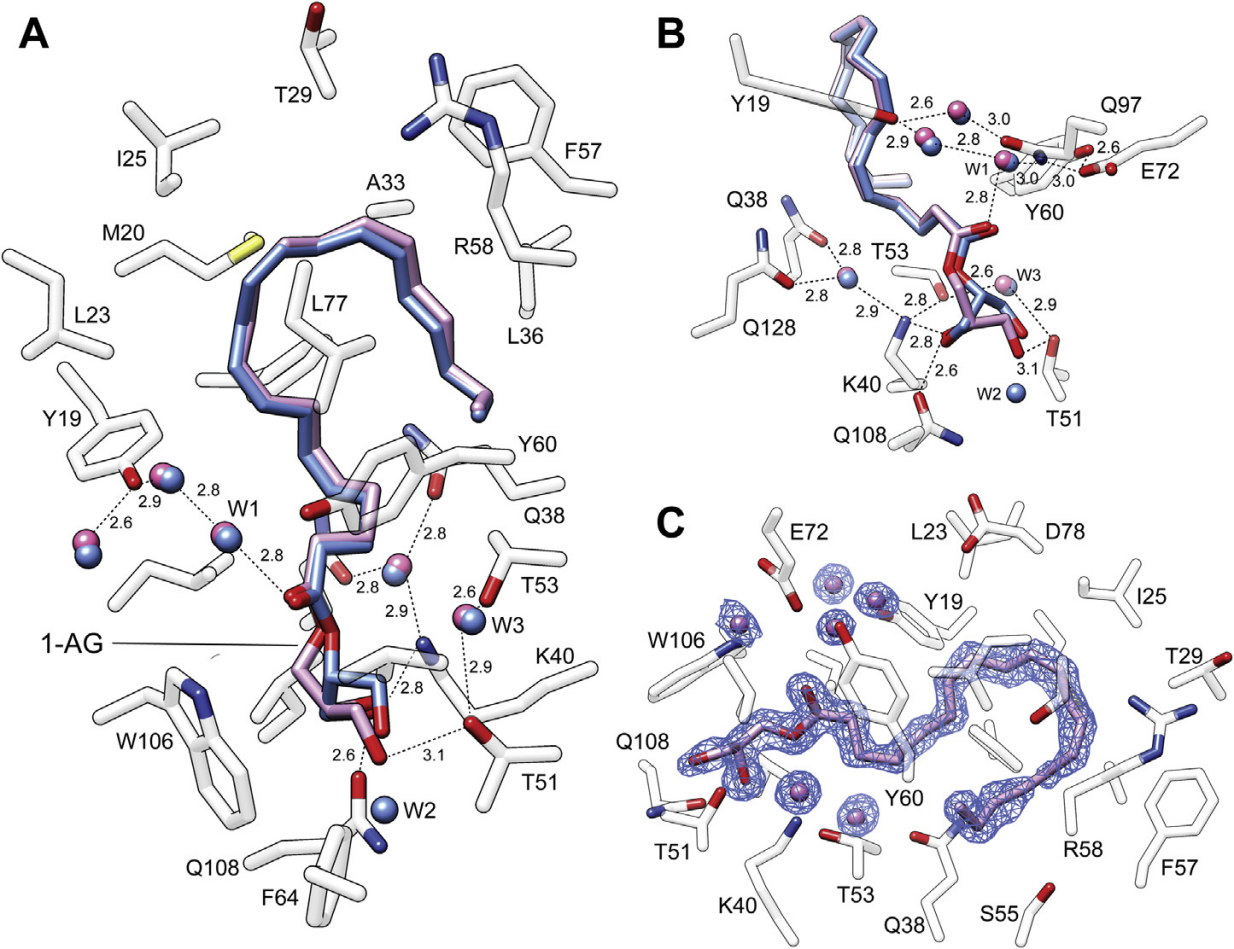
The difference in the binding modes between sn-1 and sn-2 monoacylglycerols. A: The overall position of 2-arachidonoylglycerol (blue) (PDB: 6BTH) and 1-arachidonoylglycerol (1-AG) (purple) (PDB: 7JVG) in the binding cavity. B: Close-up view of the hydrogen bond network formed by 1-AG. Ordered water molecules (W) are shown as red spheres; and dashed lines indicate hydrogen bonds. Distances are shown in angstroms. C: The 2*F*o-*F*c electron density maps for 1-AG and surrounding ordered water molecules. The map was contoured at 1.3 σ.

As evidenced by the fluoresce titrations, 1-AG is a good binding partner for CRBP2 (9). Therefore, the position of an acyl moiety in the glycerol backbone in MAGs seems not to play an important role in ligand selectivity. Indeed, the crystal structure of CRBP2 in the complex with 1-AG clearly indicates that despite geometrical differences between 2-AG and its sn-1 and sn-3 isomers, the binding modes for both molecules are identical (Fig. 4A). Inside the binding cavity, the arachidonoyl chains of 1-AG and 2-AG adapted the same orientations enforced by hydrophobic and van Der Waals interactions with the side chains of the enclosed portal region of the protein (9, 11). Also, the position of the oxygen atoms of the ester bonds were nearly overlapping (root-mean-square deviation of atomic positions, 0.3 Å), as they were locked in place by hydrogen bonds between the carboxyl oxygen and an ordered water molecule (W1) as well as the potential interaction of the ester oxygen with the ζ amino group of K40 (Fig. 4B). Similarly, the nonesterified hydroxyl groups of both ligands were engaged in the same network of hydrogen bonds that involved the side chains of K40, T51, and Q108. Thus, the identical contact points between these ligands and the protein observed for 1-AG and 2-AG could be achieved only by adapting an alternative orientation of the glycerol backbones in the binding pocket (root-mean-square deviation value for the glycerol carbon atoms = 1.3 Å). The only consequence of this difference is the 1 Å shift of the sn-3 hydroxyl group toward the location normally occupied by an ordered water (W2) in the 2-AG/CRBP2 complex. Thus, this water molecule is no longer present in the structure of 1-AG (Fig. 4B). Importantly, the binding of MAGs by CRBP2 is restricted to sn-1 and sn-2 isomers. Despite the use of a racemic mixture of 1-AG and 3-AG in the crystallographic experiments, the electron density for the ligand clearly indicated the exclusive presence of 1-AG (*S*-enantiomer) in the binding pocket (Fig. 4C).

### The effect of hydrogen bonding on the mode of ligands' binding

**Fig. 5:**
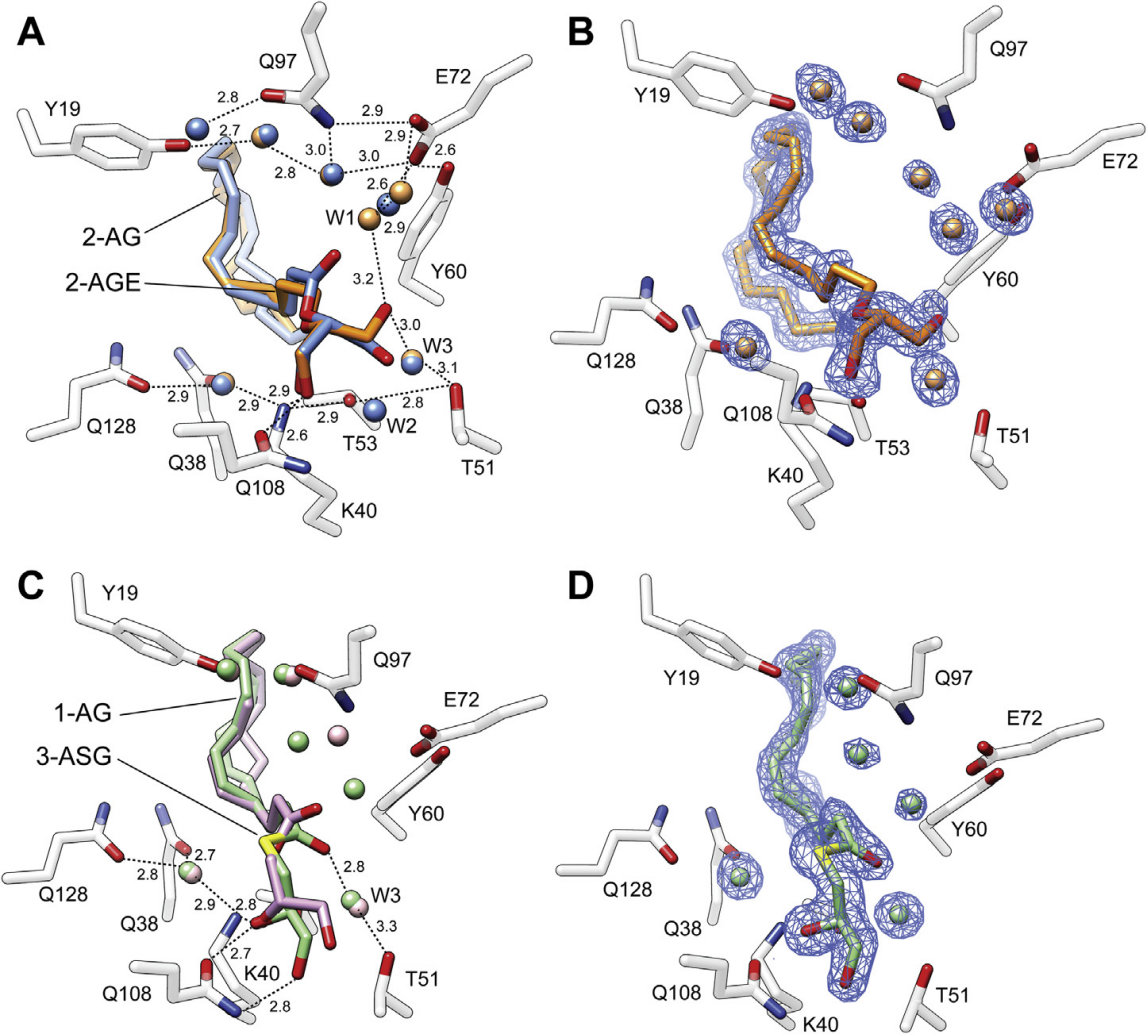
Rearrangement of the hydrogen bonding networks upon binding of monoacylglycerol derivatives. A: Comparison of the spatial orientation of 2-arachidonoylglycerol (2-AG) (blue) and its derivative lacking the carbonyl oxygen, 2-arachidonoyl glycerol ester (2-AGE, colored orange). B: The 2*F*o-*F*c electron density maps for 2-AGE and surrounding ordered water molecules. The map was contoured at 1.3 σ. C: Superposition of 1-arachidonoylglycerol (1-AG) (purple) and its derivative, arachidonoyl-3-thio-glycerol (3-ASG), in which carboxyl oxygen was replaced by a sulfur atom (thioester). 3-ASH is colored purple. D: The 2*F*o-*F*c electron density maps for 1-ASG and surrounding ordered water molecules. The map was contoured at 1.3 σ. In all panels, the ordered water molecules (W) are shown as red spheres and colored in the fashion corresponding to the ligands; dashed lines indicate hydrogen bonds. Distances are shown in angstroms.

The high affinity of MAGs for CRBP2 can be attributed to the evolvement of the glycerol backbone in the extended hydrogen bonding network inside the binding pocket. Regardless of the characteristics and position of an acyl chain, both the hydroxyl groups on MAGs are involved in similar hydrogen bonding interactions (Figs. 3 and 4). One of the hydroxyls is placed in the proximity of K40 and Q108 forming hydrogen bonds with the ζ-nitrogen and ε-oxygen atoms of these side chains (Fig. 5A). The other hydroxyl group interacts with the γ-hydroxyl of T51 and an ordered water molecule (W3). Both contact sites are part of a larger network of hydrogen bonds that also involve the neighboring side chains of Q38 and Q128. Moreover, the carbonyl oxygen of the ester bond is independently involved in hydrogen bonding via an ordered water molecule (W1) with the side chains of E72 and Q97. Probably less profound, but plausible, is another interaction site between the ester oxygen atom and the amino group of K40. The significance of these interactions is underscored by the dramatically lowered affinity of AEA, which contains only one hydroxyl group (9).

To further delineate the importance of hydrogen bonding for ligand binding, we crystallized CRBP2 with two 2-AG derivatives: 2-AGE, which lacks the carbonyl oxygen and 1-ASG, in which the ester group is substituted with a thioester bond (Table 2). Two-fold increase in the *K*_d_ value calculated for 2-AGE as compared with 2-AG is not nearly as profound as the ∼25-fold change seen for AEA (Table 1). The reason for this relatively modest effect is the ability of the remaining hydroxyls to preserve a large network of hydrogen bonding in the binding site (Fig. 5A, B). Interestingly, the crystal structure of CRBP2 in complex with 2-AGE revealed the repositioning of the hydroxyl group that no longer made direct contact with T51 as in 2-AG. Instead, it adapted an alternative orientation that allowed for its contribution to the hydrogen bond network involving the E72 and Q97 side chains. Thus, in the absence of the carbonyl oxygen, the contact made by this atom in 2-AG is substituted by the hydroxyl of 2-AGE, limiting the impact on the affinity of this lipid (Fig. 5A, B).

Analogically, the substitution of the ester group with a thioester bond had a modest effect on binding, causing a ∼2-fold increase of the *K*_d_ value for 1-ASG/arachidonoyl-3-thioglycerol (3-ASG). However, an analysis of the spatial position of this lipid in the binding site of CRBP2 revealed significant alterations in the mode of binding as compared with 1-AG. To avoid the thermodynamically unfavorable interaction of the larger and more hydrophobic sulfur atom with polar side chains in the binding site, the thioglycerol moiety of 1-ASG/3-ASG adapted a different position than the glycerol backbone of 1-AG (Fig. 5C, D). Consequently, the carboxyl oxygen was no longer part of the E72 and Q97 hydrogen network. Instead, it forms an interaction with an ordered water molecule (W3) located in between the side chain of T51 and T53. The position of a hydroxyl group involved in the interaction with K40 remained the same as in 1-AG. The second hydroxyl was positioned closer to the side chain of Q108 forming a hydrogen bond with this residue, unlike in 1-AG or 2-AG where the primary contact was with T51 (Fig. 5C). Although subtle, these alterations of the ligand's orientation resulted in the reversal of the enantioselectivity of ligand binding. In contrast to 1-AG/3-AG, which was found to exist in the binding pocket as *S*-enantiomer (1-AG), the electron density for 1-ASG/3-ASG provided evidence for the exclusive presence of *R*-enantiomer (3-ASG) (Fig. 5D).

### Ligand specificity is determined by subtle changes in the lining of the binding cavities of CRBPs

**Fig. 6:**
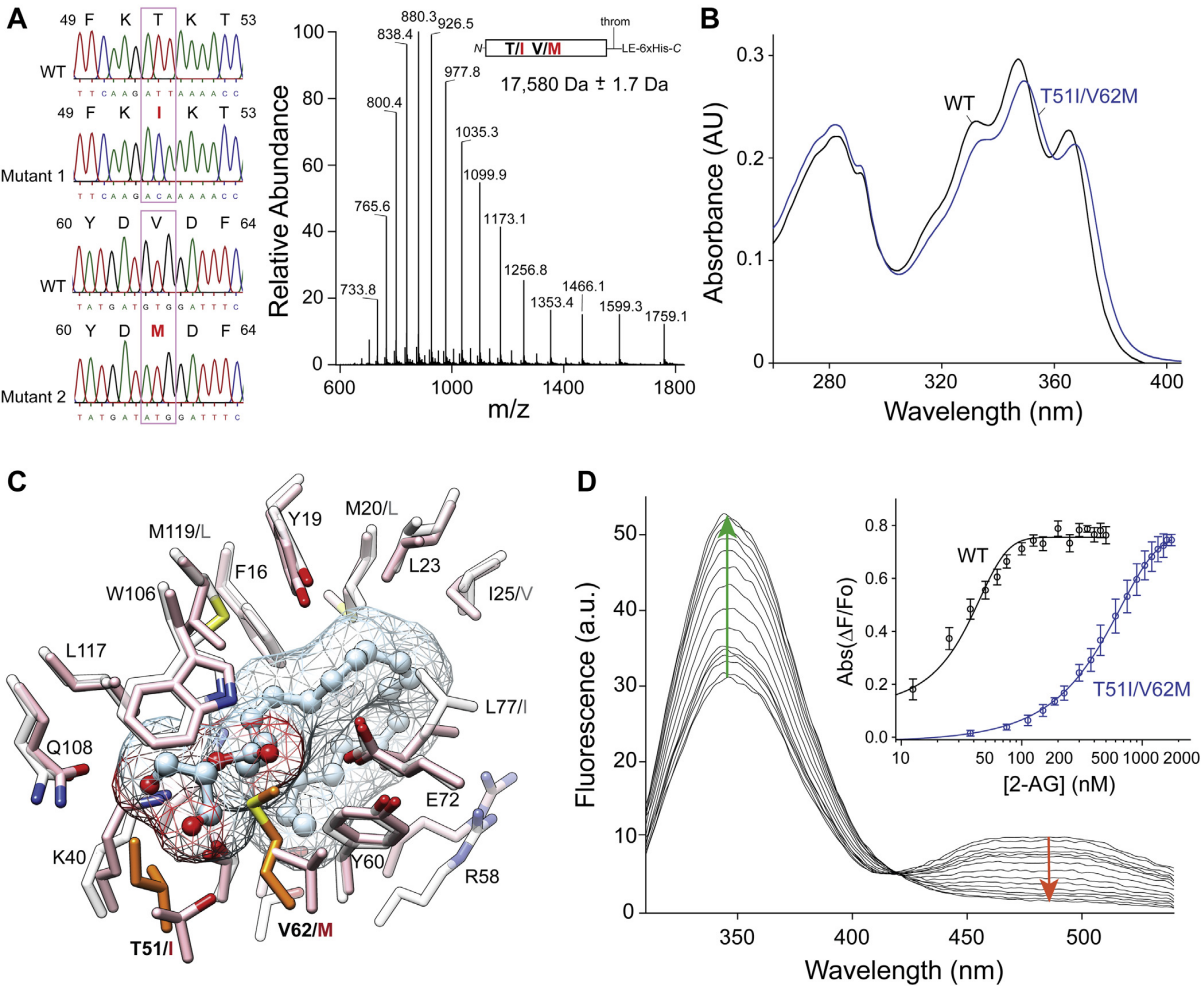
Functional characterization of cellular retinol binding protein 2 (CRBP2)^T51I/V62M^ mutant. A: Sequencing of mutated CRBP2 cDNA indicated the intended substitutions. The right panel represents mass spectrum of intact CRBP2^T51I/V62M^. The deconvoluted molecular weight of this protein was identical to that calculated based on the amino acid sequence. B: UV/visible absorbance spectra of WT and mutated CRBP2. The spectral characteristic between 330 and 380 nm indicated formation of the protein-atROL complex. C: Location of the mutated residues within the binding cavity of cellular retinol binding protein 1 (gray) and CRBP2 (pink). Residues I51 and M62 present in the cellular retinol binding protein 1 are colored orange. 2-Arachidonoylglycerol (2-AG) molecule present in the binding pocket is represented by ball and stick. D: Fluorescence spectra from CRBP2^T51I/V62M^ titrated with increasing concentrations of 2-AG. Direction of the changes in the fluorescence signal for the protein scaffold and the retinoid moiety are marked with green and red arrows, respectively. The titration curves for WT and CRBP2^T51I/V62M^ variant reveal a dramatic shift in the *K*_d_ values for the mutated protein (inset).

Similar molecular architecture does not imply identical affinity and lipid selectivity in CRBPs. Differences in ligand binding characteristics were initially observed for affinities to atROL (23). They most likely also contribute to the selectivity of CRBPs for other lipid molecules. For example, the tight binding of atROL by CRBP1 (*K*_d_ ∼10 nM) corresponds with the exclusive interaction of this protein with retinoids, whereas the lower affinity of CRBP2 for atROL appears to correlate with selectivity for MAGs. To reconcile the differences in the binding specificity between these two proteins, we overlapped the spatial orientation of 2-AG bound to CRBP2 with the structure of CRBP1 and compared the amino acid composition in the direct vicinity of the ligand. As expected based on the high sequence homology (67% identity and 89% similarity), the overall architecture of the binding sites is nearly identical between CRBP1 and CRBP2 (Fig. 6). However, we identified two residues in CRBP1 (I51 and M62) that potentially prevent interaction with MAGs. To provide experimental evidence for the involvement of I51 and M62 in narrowing ligand specificity, we generated a CRBP2 mutant protein in which T51 and V62 were substituted with isoleucine and methionine residues, respectively (Fig. 6A). The purified mutant protein (CRBP2^T51I/V62M^) was fully functional as evidenced by its ability to form a stable complex with atROL, the spectral characteristic of which closely resembled that of native CRBP2 (Fig. 6B). However, the examination of the binding of 2-AG revealed an over 16-fold decrease in the affinity for this ligand signified by a *K*_d_ value of 447.3 ± 46.1 nM (Fig. 6D). The observed decline in the affinity for 2-AG can be explained mechanistically by a combination of three factors: *i*) the elimination of the hydrogen bonds between the hydroxyl group of the ligand and the threonine side chain, *ii*) the increased hydrophobicity of the binding site in close proximity to the polar hydroxyl group of the glycerol backbone of the ligand, and *iii*) the steric hindrance introduced by the large side chain of methionine (Fig. 6C). Altogether, we provided evidence that ligand selectivity can be switched between members of the CRBP family by relatively subtle substitutions in key residues within the binding cavity.

## Discussion

In addition to the binding of atROL, our data unambiguously establish the preferential interaction of CRBP2 with MAGs. The binding of MAGs is an intrinsic property of CRBP2 that distinguishes it from CRBP1 and probably other members of the CRBP protein subfamily. The ability to interact with MAGs is highly specific and arises for definite structural reasons. It is enabled by the substitution of just two amino acids (T51I and V62M) within the binding site of CRBP2 as compared with exclusively retinoid binding CRBP1. This observation proves the extraordinary versatility of the lipocalin scaffold that can be easily modified to accept diverse lipophilic ligands. Importantly, these gain-of-function modifications in the architecture of the binding site broadened but did not eliminate the relatively narrow ligand specificity of CRBP2. The highest affinity for sn-1 and sn-2 MAGs with C18-C20 polyunsaturated fatty acid chains is achieved by a combination of the discrete hydrogen bond network formed by the ligand inside the binding pocket and its hydrophobic and van der Waals contacts with the amino acids of the portal region. Occurring simultaneously, these two kinds of interactions define the chemical boundaries for ligands that can form a stable complex with CRBP2. Altering just one of these interactions leads to the partial loss of affinity or failure to stabilize the protein in its ligand-bound close conformation.

An open question remains whether CRBP2 is the only member of the CRBP protein family that can interact with nonretinoid ligands. A comparison of the primary sequence and the architecture of CRBP3 and CRBP4 does not provide a direct answer. Even though human CRBP3 contains a valine residue in the position 62 like CRBP2, this amino acid is not conserved across different species, being substituted with a methionine in the *Xenopus tropicalis* and chicken orthologues. Moreover, the T51 residue conserved in CRBP2 is replaced by valine, which makes the CRBP3 binding site comparable to CRBP1. Similarly, CRBP4 contains a preserved isoleucine residue in position 51 like CRBP1. However, M62 is substituted by valine, which is characteristic for CRBP2. The possibility that CRBP3 and CRBP4 may selectively bind a subset of nonretinoid lipids is supported by the fact that the affinity for atROL calculated for these proteins is orders of magnitudes lower than for CRBP1. Also, despite the availability of high-resolution crystal structures of these proteins, none of them has been crystallized in complex with a retinoid ligand (24, 25). Moreover, the metabolic phenotype of CRBP3-deficient mice that included a lower susceptibility to dyslipidemia and obesity when fed a high-fat diet may suggest the existence of an alternative to atROL endogenous ligands for this protein (26). The clarification of the ligand specificity of CRBPs would be an important step in the elucidation of the physiological roles of this class of lipid carrier proteins.

The dual ligand selectivity of CRBP2 raises a key question about the role of this protein in the facilitation of retinoid and MAG absorption and metabolism. Undoubtedly, CRBP2 plays a pivotal role in the optimal dietary uptake of retinoids particularly in times of dietary vitamin A insufficiency (8, 27). However, the induction of jejunum *Rbp2* expression in rats fed a high-fat diet as well as an elevated mucosal level of MAGs and systemic metabolic disorders observed in CRBP2-deficient mice indicate the involvement of this protein in metabolism and/or neutral lipid signaling (9, 28). The exact mechanism responsible for the modulation of lipid metabolism by CRBP2 remains to be elucidated. The ability of MAGs to outcompete atROL for the binding site may suggest an adverse impact on the efficiency of retinoid absorption resulting from dietary fat intake. However, a high abundance of CRBP2 in enterocytes and the presence of other lipid binding proteins, such as fatty acid binding protein 1 (FABP1) may prevent direct competition between retinoids and MAGs (29, 30, 31). Moreover, unlike the promiscuous FABP1, CRBP2 revealed surprising selectivity toward polyunsaturated MAGs, with the highest affinity for 2-AG. Thus, it is most likely that CRBP2 does not play a redundant role to FABP1 and contribute to bulk lipid uptake but rather facilitates the biological activity of a subclass of signaling MAGs. This stipulation is further supported by the well-documented link between intestinal endocannabinoid signaling via cannabinoid receptors and GPCR119 on energy expenditure and body weight (32, 33). The impaired metabolic phenotype of *Rbp2*^−/−^ mice is associated with elevated levels of 2-AG. Signaling through GPCR119, this endocannabinoid contributes to the synthesis and secretion of GIP from the enteroendocrine K cells, the concentration of which was two times higher in *Rbp2*^−/−^ mice after gavage with corn oil as compared with WT animals (9, 34). Importantly, the elevated levels of GIP have been reported in diet-induced obese mice as well as patients with type 2 diabetes (35, 36). Thus, obesity, glucose intolerance, and fatty liver phenotypes documented in *Rbp2*^−/−^ mice might arise as a consequence of an increased concentration of 2-AG and other bioactive MAGs. Alternatively, this may involve a retinoid-mediated effect resulting from a combination of both MAG and retinoid actions. This will require further research to elucidate.

There is no doubt that many more biochemical, genetic, endocrine, and nutritional studies need to be done to fully dissect the contribution of CRBP2 to intestinal and systemic lipid homeostasis. Nevertheless, the determination of the ligand selectivity of this protein and an understanding of the biochemical principles that govern lipid binding at the molecular level provide important knowledge that will help focus future experiments.

## Data availability

The structure factor files and the corresponding models of CRBP2 complexes with lipid ligands described in this article have been deposited in PDB under the following accession numbers: 7JVG, 7JVY, 7JWD, 7JWR, 7K3I, 7JX2, and 7JZ5.

## Supplemental data

This article contains supplemental data.

## Conflict of interest

The authors declare that they have no conflicts of interest with the contents of this article.

## Supplemental data

Supplement al Table S1 and Fig. S1
