# Supplementary material for: Molecular basis for the interaction of cellular retinol binding protein 2 (CRBP2) with nonretinoid ligands
Source: J Lipid Res. 2021 Feb 23;62:100054. doi: 10.1016/j.jlr.2021.100054 (PMC8010219; doi:10.1016/j.jlr.2021.100054)
Supplement: Supplement — al Table S1 and Fig. S1 [file mmc1.docx]

**SUPPLEMENARY MATERIALS**

**Molecular basis for the interaction of cellular retinol-binding protein 2 (CRBP2) with non-retinoid ligands**

**Josie A. Silvaroli^1#^, Jacqueline Plau^1#^, Charlie H. Adams^1^, Surajit Banerjee^2, 3^, Made Airanthi K. Widjaja-Adhi^1^, William S. Blaner^4^, and Marcin Golczak^1, 5*^**

From the ^1^Department of Pharmacology, School of Medicine, Case Western Reserve University, Cleveland, OH

^2^Department of Chemistry and Chemical Biology, Cornell University, Ithaca, NY;

^3^Northeastern Collaborative Access Team, Argonne National Laboratory, Argonne, IL;

^4^Departments of Medicine1, College of Physicians and Surgeons, Columbia University, New York, NY

^5^Cleveland Center for Membrane and Structural Biology, School of Medicine, Case Western Reserve University, Cleveland, OH

^*^To whom the correspondence should be addressed: Marcin Golczak, Ph.D., Department of Pharmacology, School of Medicine, Case Western Reserve University, 10900 Euclid Ave, Cleveland, Ohio 44106, USA; Phone: 216–368–0302; Fax: 216–368–1300; E–mail: mxg149@case.edu.

^#^these authors contributed equally to this work

**Supplementary Table 1** – Classes of lipids represented in Enzo and Cayman Chemical libraries used in the study.

| LIPID CLASS | ENZO  SCREEN-WELL Bioactive | CAYMAN  Bio-active Lipid I |
| --- | --- | --- |
| Endocannabinoids | X | X |
| Farnesyl/geranylgenaryl derivatives | X | X |
| Hydro- and dehydroxyeicosatetraenoic acids | X |  |
| Hepoxilins | X |  |
| Polyunsaturated fatty acids | X | X |
| Leukotrienes | X | X |
| Lipoxins | X |  |
| Phosphatidic acids/lysophosphatidic acids | X | X |
| Octadecanoids | X | X |
| Platelets activation factors | X | X |
| Prostaglandins/thromboxanes | X | X |
| Retinoids | X | X |
| Vitamin D metabolites | X |  |
| Sphingolipids | X | X |
| D-myo-inositol-phosphates |  | X |
| Phosphatidylinositol-phosphates |  | X |
| Ceramide derivatives |  | X |
| Lipid-based receptor agonists and antagonists | X | X |
| Lipid-like drugs and inhibitors | X | X |

**
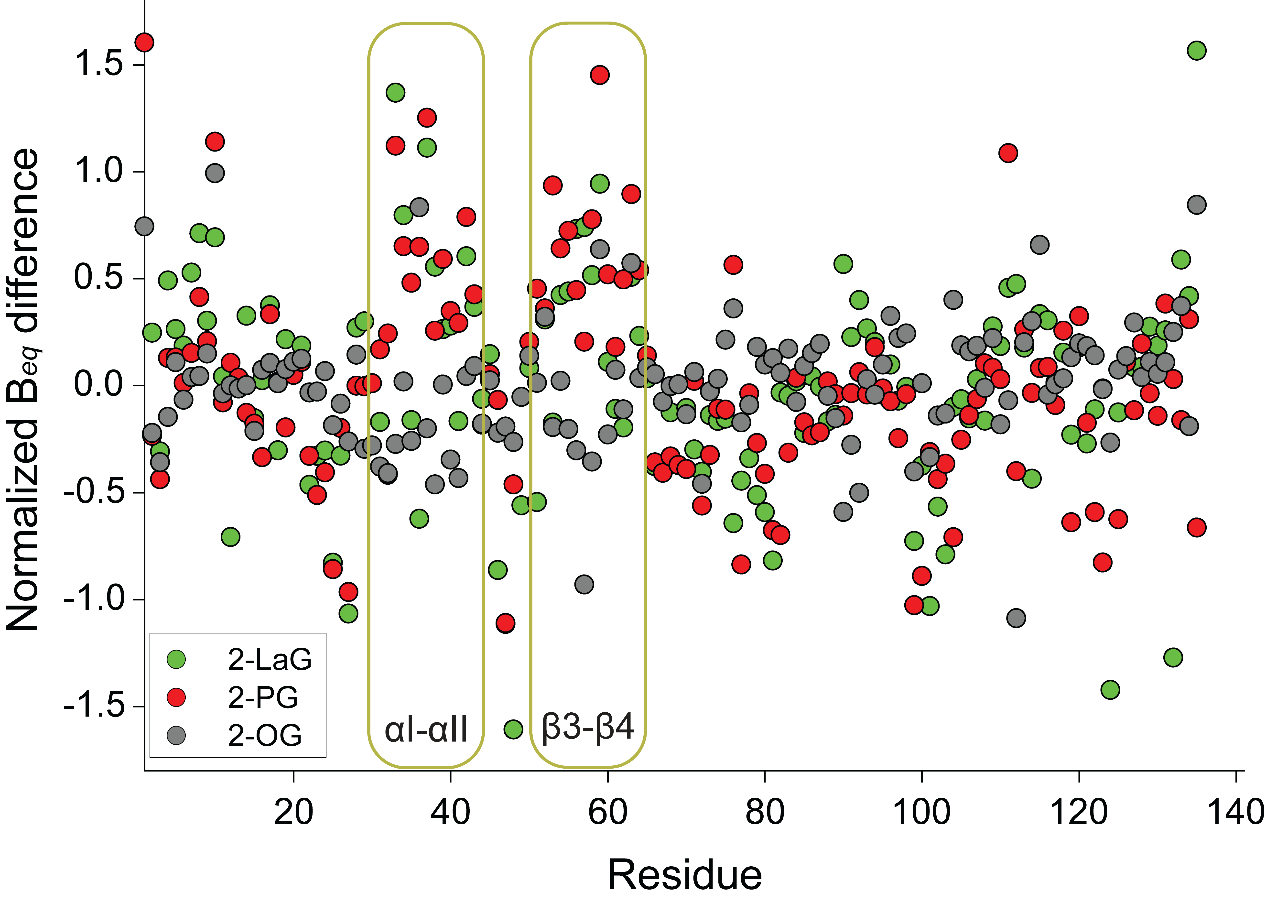
**

**Supplemental figure 1** – *Differences in the equivalent isotropic B-factors between CRBP2 in complex with 2-AG and CRBP2 bound to 2-LaG, 2-PG, and 2-OG.* The plot represents relative differences in normalized B*_eq_*. and indicates regions with increased conformational dynamics.
